# Vectors as Epidemiological Sentinels: Patterns of Within-Tick *Borrelia burgdorferi* Diversity

**DOI:** 10.1371/journal.ppat.1005759

**Published:** 2016-07-14

**Authors:** Katharine S. Walter, Giovanna Carpi, Benjamin R. Evans, Adalgisa Caccone, Maria A. Diuk-Wasser

**Affiliations:** 1 Department of Epidemiology of Microbial Diseases, Yale School of Public Health, New Haven, Connecticut, United States of America; 2 Department of Ecology and Evolutionary Biology, Yale University, New Haven, Connecticut, United States of America; 3 Department of Ecology, Evolution, and Environmental Biology, Columbia University, New York, New York, United States of America; Monash University, AUSTRALIA

## Abstract

Hosts including humans, other vertebrates, and arthropods, are frequently infected with heterogeneous populations of pathogens. Within-host pathogen diversity has major implications for human health, epidemiology, and pathogen evolution. However, pathogen diversity within-hosts is difficult to characterize and little is known about the levels and sources of within-host diversity maintained in natural populations of disease vectors. Here, we examine genomic variation of the Lyme disease bacteria, *Borrelia burgdorferi* (*Bb*), in 98 individual field-collected tick vectors as a model for study of within-host processes. Deep population sequencing reveals extensive and previously undocumented levels of *Bb* variation: the majority (~70%) of ticks harbor mixed strain infections, which we define as levels *Bb* diversity pre-existing in a diverse inoculum. Within-tick diversity is thus a sample of the variation present within vertebrate hosts. Within individual ticks, we detect signatures of positive selection. Genes most commonly under positive selection across ticks include those involved in dissemination in vertebrate hosts and evasion of the vertebrate immune complement. By focusing on tick-borne *Bb*, we show that vectors can serve as epidemiological and evolutionary sentinels: within-vector pathogen diversity can be a useful and unbiased way to survey circulating pathogen diversity and identify evolutionary processes occurring in natural transmission cycles.

## Author Summary

Lyme disease, caused by a bacteria carried by deer ticks, is the most common vector-borne disease in North America and over 30,000 cases are reported each year in the United States. Ticks may be infected with multiple strains of the Lyme disease bacteria, which differ in transmissibility and the harm they pose to humans. In this study, we collected 98 infected deer ticks from across the United States and southern Canada. We used genetic techniques to investigate the diversity of the Lyme disease bacteria infecting each individual tick. We find that 70% of ticks are infected with multiple strains of the Lyme disease bacteria, indicating that humans may be exposed to and infected with multiple bacterial strains from a single tick bite. We also find evidence that the Lyme disease bacteria is evolving in response to the immune defenses of its natural hosts (including rodents and birds). Our study shows that individual ticks and other disease vectors can be studied as epidemiological sentinels, which reveal the extensive diversity of pathogens circulating in natural disease cycles and how they are evolving.

## Introduction

Hosts including humans, other vertebrates, and arthropods, are frequently co-infected with multiple pathogen species in addition to diverse populations of pathogens of the same species. Within-host pathogen diversity may have important implications for human health and disease epidemiology as complex infections may differ in virulence, antibiotic susceptibility, and transmissibility[1–4]. The ecological dynamics of diverse within-host pathogen populations—including competition and facilitation—may drive pathogen evolution, including the evolution of virulence[3,5,6]. Within-host competition (through exploitation of host resources, host immune-mediated apparent competition, or direct interference) selects for pathogen strains that are the best within-host competitors; if within-host competitive success is linked to virulence, within-host diversity may drive virulence evolution [7]. Neutral and adaptive evolutionary processes acting on heterogeneous pathogen populations, including population bottlenecks occurring during the host’s infection and during transmission[8,9], selection within and between-hosts [9–11], and recombination between co-infecting strains[12] further shape patterns of pathogen diversity.

Within-host diversity may arise from multiple independent infection events, pre-existing diversity in the inoculum, and *in situ* evolution. Distinguishing between these sources of diversity allows us to identify ecological and evolutionary processes occurring within-host (e.g. within-host competition of strains and/or host-imposed selection) or across hosts (e.g. transmission dynamics and/or population-level selection)[3].

**Fig 1:**
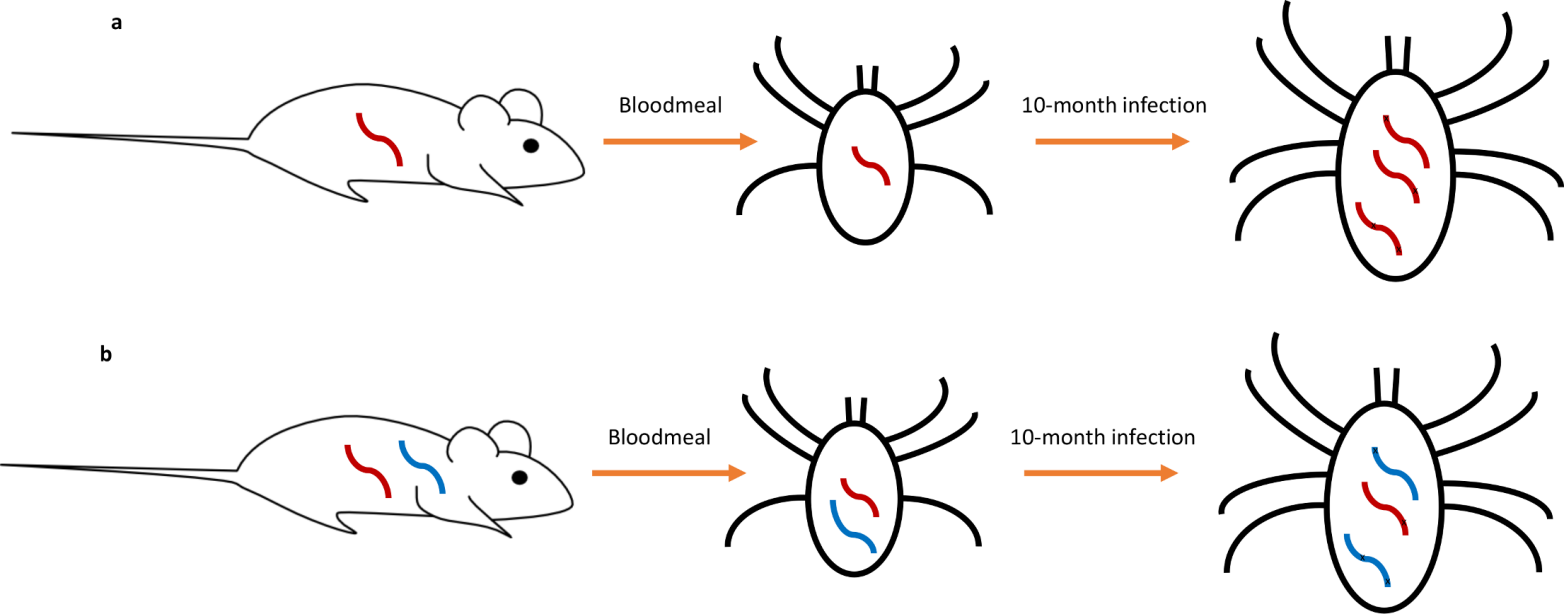
*Bb* diversity in nymphal ticks is derived from two sources. (a) Larval ticks may be infected with a single *Bb* strain that may undergo *in situ* evolution over the course of the tick’s ten-month infection, a single infection. (b) Alternatively, larval ticks may be infected with a diverse infecting inoculum from the vertebrate host and the mixed-strain *Bb* population may further undergo *in situ* evolution over the tick’s 10-month infection. *De novo* mutations acquired over the ticks’ infection are represented by **x**’s.

*Ixodes scapularis* tick vectors of the Lyme disease spirochete, *Borrelia burgdorferi* (*Bb*), offer a unique model for study of within-host pathogen diversity. Infected nymphal ticks are a simplified host compared to chronically infected humans, the focus of most studies of within-host pathogen diversity. Humans may harbor superinfections from an unknown number of infection events occurring at unknown times in the past [2,10]. Pathogen samples from humans, including tissue, sputum, or cultured samples may be biased, as only a few strains may be represented in a sample[13]. In contrast, infected nymphal ticks are infected with a single inoculum (infecting bloodmeal) as larvae (because *I*. *scapularis* feed only once per life stage, although[14,15]**).** Further, the *I*. *scapularis* lifecycle is strongly seasonal[16,17]. Therefore, we can estimate the time elapsing between the larval bloodmeal and nymphal host-seeking activity (time of tick sampling), which corresponds to the duration of tick infection [18,19]. With no opportunity for superinfection, within-tick diversity is derived from only two sources: *in situ* evolution over the course of the ticks’ infection and diversity present in the inoculum (Fig 1). Further, sampling pathogens directly from an entire tick (i.e. not a sample of tick tissue) captures a large spectrum of pathogen diversity, since detection of within-tick variation is theoretically limited only by sequencing depth.

Genomic approaches provide a lens to peer into pathogen diversity at an unprecedented level, revealing that pathogen infections are comprised of heterogeneous populations [20]. However, sampling and bioinformatic challenges make it difficult to assess patterns of within-host pathogen variation[20] and most population deep sequencing studies of within-host diversity have focused on rapidly evolving RNA viruses. Previous studies of within-vector pathogen diversity have focused on deep sequencing of single locus or a set of loci[21,22], clonal isolates[11,22], or empirically-derived samples[23,24]. This is, to our knowledge, the first study to characterize pathogen diversity present within field-collected disease vectors using population whole-genome sequencing data.

Here, we quantify diversity of the Lyme disease bacterium, *Bb*, within and across individual field-collected tick vectors. For each tick, we assess support for two alternative mechanisms generating observed within-tick variation: (a) *in situ* evolution and (b) mixed strain infection. We determine the prevalence of mixed infections and examine the ecological and evolutionary (selective) processes shaping *Bb* diversity within and across ticks.

## Results and Discussion

### Sampling natural populations of *B. burgdorferi* vectors

We sampled 98 infected ticks from across the northeastern and midwestern invasion foci as questing nymphal ticks (seeking bloodmeals from vertebrate hosts) (S1 Fig, S1 Table). Samples were collected over a 15-year sampling period (1998–2013, S1 Fig). Nymphal ticks feed on all available vertebrate hosts and provide a snapshot of population-wide *Bb* diversity. We captured *Bb* DNA directly from whole tick genomic DNA extracts, using a hybrid capture and deep sequencing approach we previously developed[25].

### Within-tick variant identification

**Fig 2:**
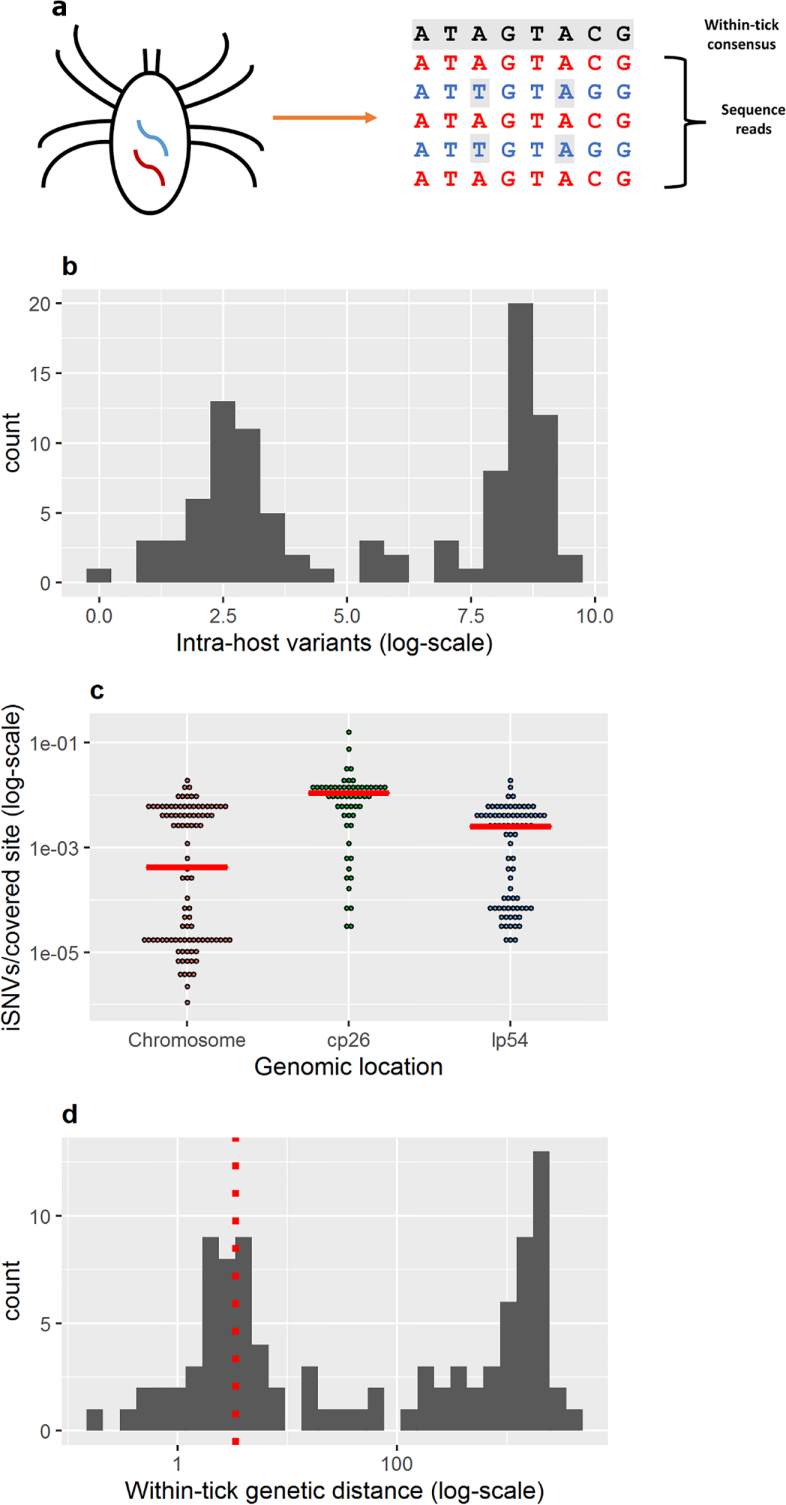
Within-tick *Bb* diversity across ticks. (a) Deep sequencing reveals within-tick *Bb* variation. We generate a within-tick consensus *Bb* sequence for each host, map sequence reads to the consensus sequence, and identify iSNVs (indicated in grey). (b) Number of *Bb* iSNVs across individual ticks. Histogram bars represent the count of individual ticks harboring total *Bb* diversity falling within the iSNV bin on the x-axis. (c) The normalized number of *Bb* iSNVs per site with > 40 X coverage (60% iSNV calling power for minor alleles comprising 10% of the population, S1 Text). No significant differences exist in iSNV rates between chromosome and plasmids (Mann-Whitney test, p-value > 0.1). (d) Genetic distance for the *Bb* population infecting each tick. Genetic distance is calculated as the sum of the minor allele frequencies across the *Bb* genome. The red dotted line indicates the estimated threshold above which ticks are classified as harboring “mixed” infections.

Identifying low-frequency intrahost single-nucleotide variants (here, iSNVs) is challenging because biological sequence variation must be distinguished from errors introduced through genomic sequencing or mapping[20]. We constructed consensus genomes for the *Bb* population infecting each tick, mapped reads to the tick’s consensus *Bb* genome, and identified iSNVs with a set of conservative filters (Methods) (Fig 2A). Generating a consensus genome for the *Bb* population infecting each tick (a conservative estimate of the majority strain) allowed us to measure genetic distance from the consensus sequence (and test hypotheses about the source of within-tick diversity) and predict the biological consequence of mutations between the two (or more) circulating *Bb* strains infecting the tick.

To test the specificity and sensitivity of our pipeline for detecting true biological iSNVs, we simulated both single and mixed infections of *Bb in silico* (S1 Text) and found that our approach is both highly specific, > 99.9% of iSNVs detected are true iSNVs, and sensitive, ~60% for individual iSNVs present at minor allele frequencies (MAF) of 10%, (S2 Fig, S3 Fig, S1 Text).

### What levels of within-tick Bb diversity occur in field-collected ticks?

Deep population sequencing reveals both extensive *Bb* variation within individual infected ticks and variation in the magnitude of within-host diversity across ticks: we detect 0 to 13,000 *Bb* iSNVs per tick (Fig 2B). Controlling for sequencing depth, this corresponds to a median of 2.9 x 10^−4^ iSNVs/covered site (Fig 2C), which we define as sites with > 40X coverage (S1 Text). The observed rate of within-tick *Bb* variation is comparable to that of Lassa and Ebola viruses in human hosts[26] and higher than that of the bacterial pathogen *Burkholderia dolosa* in chronically infected human hosts[10]. In contrast to the above examples, in which the majority of within-host diversity is generated through *de novo* mutations occurring during the sampled host’s infection, infected ticks frequently hold a sample of *Bb* pre-existing diversity (Fig 1).

To test if the level of within-tick *Bb* diversity is consistent with *in situ* evolution (a single infection) or if *Bb* diversity must have been present in the infecting bloodmeal (a mixed infection), we determine the maximum genetic diversity consistent with *in situ* evolution from a single infecting strain (Methods). Infected nymphal ticks have taken only a single bloodmeal (as larval ticks) at the time of infection and they exhibit strongly seasonal feeding behavior[16,17]. The duration of tick infection is therefore the time from the larval bloodmeal to the nymphal bloodmeal, ~ 340–375 days (Fig 1, S4 Fig, and Methods). Given a conservatively fast estimate of *Bb* mutation rate, we can determine a maximum threshold for *Bb* diversity (i.e. genetic distance) possible due to *in situ* evolution away from a single infecting *Bb* strain: 1.03 mutations. Because sequencing and mapping errors may introduce false within-tick variants, we additionally estimate a sequencing/mapping error threshold above which true biological within-tick variants can be distinguished from sequencing/mapping noise (Methods, Text S1). A genetic distance greater 3.15, the sum of the maximum genetic distance due to *in situ* evolution (1.03 mutations) and the sequencing/mapping error threshold (2.33 mutations) constitutes evidence of a mixed infection.

Within-tick *Bb* genetic distance is 0–4000 mutations (Fig 2D). The majority of ticks, 69.4% (68/98), harbor a mixed strain *Bb* infection (i.e. levels of *Bb* diversity pre-existing in the infecting inoculum).

**Fig 3:**
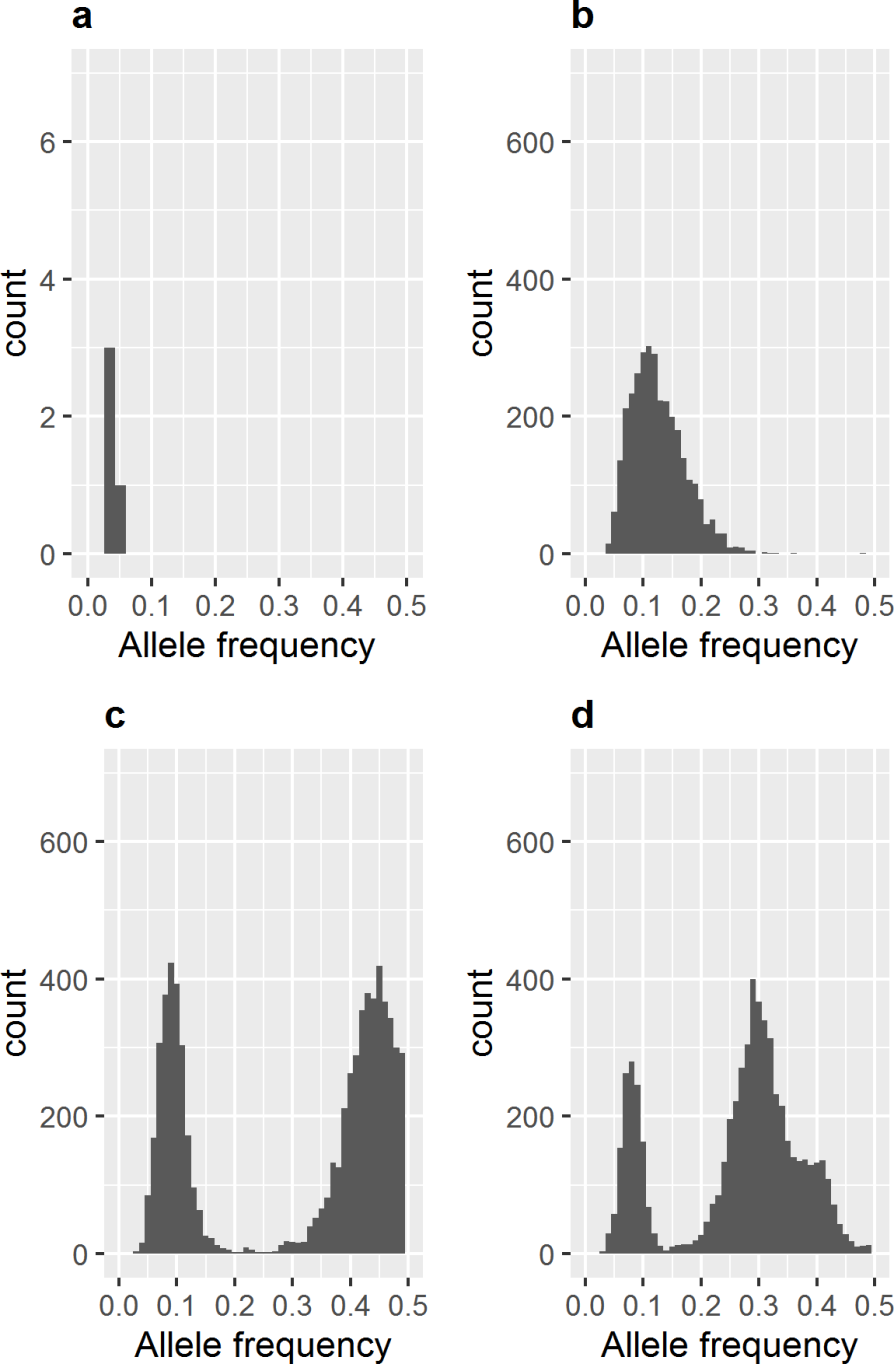
Within-tick *Bb* diversity in single infected ticks. (a) The minor allele frequency (MAF) spectrum of a representative “singly” infected tick sample. The *Bb* population infecting Bbcap22 (600 X coverage) harbors few variants with MAF < 5%, likely accrued via *in situ* evolution. Histogram bars represent the count of genomic sites with a minor allele frequency that falls within the MAF frequency bin on the x-axis. Note the differing y-axes for (a) and (c, d, and e). (b) MAF spectrum of a “multiply” infected tick sample, Bbcap5 (109 X coverage). The *Bb* population infecting Bbcap5 harbors a high number of intermediate frequency minor alleles, a level of diversity likely preexisting within a diverse inoculum. (c) MAF spectrum of a “multiply” infected tick sample, Bbcap17 (221 X coverage). (d) MAF spectrum of a “multiply” infected tick sample, Bbcap31 (309 X coverage).

Starkly different patterns of *Bb* diversity within individual ticks suggest multiple evolutionary mechanisms shaping observed *Bb* diversity. While the representative tick in Fig 3A is characterized by few variants with a minor allele frequency < 5%, consistent with *in situ* evolution, those in Fig 3B–3D harbor high levels of within-tick diversity, likely the outcome of a complex infecting inoculum. Multiple peaks in the *Bb* minor allele frequency (MAF) distributions reveal the presence of minority strains comprising different proportions of tick’s infection. However, as minority strains may share mutations in common relative to the majority *Bb* strain, it is not possible to interpret each individual peak in the MAF distribution as an additional strain. (For example, the *Bb* population in Fig 3D may include three minority strains comprising 10%, 30%, and 40% of the total *Bb* population. Alternatively, the *Bb* population may include only two minority strains that share mutations relative to the majority strain, creating an additional peak at 40%.)

The different patterns of within-tick diversity demonstrates that multiple clonal populations of *Bb* circulate in natural populations and co-transmission of multiple clones is common. While some within-tick variation is introduced through *de novo* mutations occurring in the tick or vertebrate hosts (indicated by the iSNVs present at low frequencies), the stark peaks in the MAF distributions demonstrate that multiple clonal cohorts of *Bb* co-exist at intermediate frequencies within hosts and through transmission cycles[27]. (As stated above, individual peaks do not necessarily represent additional minority strains.) Variation around peaks is most likely due to noise in estimation of allele frequencies in addition to *de novo* mutation between minority variants. Not only is significant *Bb* diversity transmitted from vertebrate hosts to ticks, but also diversity is maintained through a predicted bottleneck in the molt from larvae to nymphs.

### What ecological processes drive the observed diversity?

To test for evidence of within-tick competition or facilitation between co-infecting *Bb* strains, we quantified differences in *Bb*-infection intensity in singly and multiply infected ticks. In the absence of interactions between co-infecting *Bb* strains, *Bb*-infection intensity should increase additively with the number of strains present[28]. Inter-strain competition would yield a lower than additive increase in the number of spirochetes with increased number of strains and vice versa. We found no significant difference between the infection intensity of singly and multiply infected samples (S5 Fig, Mann-Whitney test, p = 0.543), suggesting competition between coinfecting *Bb* strains.

To test if levels of within-tick *Bb* diversity carried a signature of the ongoing *Bb* invasion[29], we tested for associations between sampling year and sampling location and within-tick *Bb* genetic distance. We predicted that ticks sampled early in the *Bb* invasion or at the leading edge of invasion would harbor less within-tick *Bb* diversity. We found no effect of sampling year on within-tick *Bb* diversity across all 98 samples (S6A Fig, F-test, p = 0.619), nor in the 68 samples collected in the Northeast (S6B Fig, F-test, p = 0.583). Within-tick *Bb* diversity did not show significant spatial autocorrelation (i.e. sampling location was not correlated with levels of within-tick *Bb* diversity) for all 98 samples (Moran’s I, p = 0.958), nor in the 68 samples collected in the Northeast (Moran’s I, p = 0.813).

Within-tick *Bb* diversity did vary regionally. Samples collected in Virginia had significantly lower within-tick *Bb* diversity than samples from any other region (S7 Fig, Mann-Whitney test, regional comparisons of Virginia vs. Midwest, Canada, and Northeast, all p < 0.005). Reduced within-tick diversity found in Virginia samples may reflect the recent expansion of ticks at Virginia collection sites (personal communication).

### What evolutionary processes drive observed Bb diversity?

Since the majority of *Bb* iSNVs were present in a diverse inoculum, they constitute a sample of the *Bb* variation present within vertebrate hosts. Within-tick *Bb* polymorphisms reflect pathogen diversity generated at some point in the past; individual ticks thus hold a historic record of historic population-level selective processes.

We examined the role of natural selection in shaping within-tick *Bb* diversity. Within each tick with evidence of a mixed infection, we predicted the effect of each polymorphism using snpEff[30] and evaluated dN/dS ratios (the ratio of non-synonymous to synonymous amino acid mutations), the canonical measure of selection. As predicted, in ticks with mixed infections, we found strong evidence of purifying selection across the *Bb* genome (mean dN/dS = 0.15, sd = 0.17), similar to what is observed for Lassa virus iSNVs (dN/dS~0.2) [26]. The strength of purifying selection varies across the genome (S2 Table, S8 Fig).

**Fig 4:**
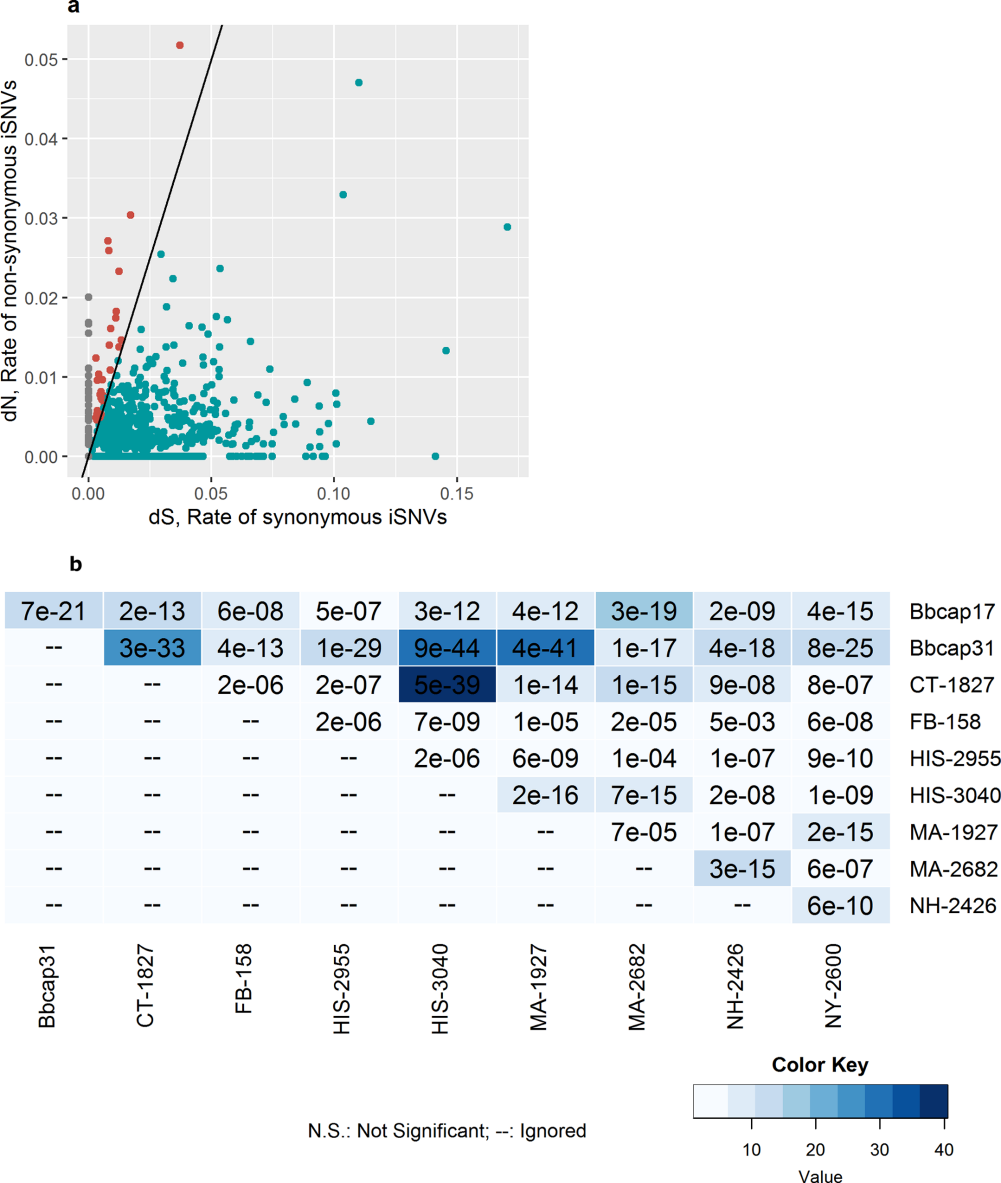
Detecting selection across the *Bb* genome. (a) Within a single tick (Bbcap17), dN plotted against dS for each of the 876 genes on the *Bb* chromosome and plasmids cp26 and lp54. Each point represents a single gene. The line represents the neutral expectation dN = dS. Red points falling in the upper left half of the plot have a signal of positive selection while blue points falling in the lower right have a signal of purifying selection. Grey points represent genes with nonsynonymous variants, but no synonymous variants, so dN/dS could not be determined. (b) Overlap between genes under positive selection (dN/dS >1) in each multiply infected tick (the 10 ticks with greatest within-tick *Bb* diversity are shown for clarity, resulting in 9 pairwise comparisons for each tick). For each pair of ticks, the odds ratio representing the strength of association between the two gene sets under positive selection is colored according to the color ramp: dark blue indicates that gene sets are strongly associated. P-values from a Fisher’s exact test of association between gene sets are super-imposed on each cell.

Within-tick dN/dS varies widely across the 876 predicted *Bb* genes on the chromosome and two plasmids, and revealing genes with a signal of positive selection. In a single tick, for example the tick in Fig 4A, the 29 *Bb* genes with a signal of positive population-level selection include surface exposed proteins *GlpE* and *P13*. Because mutations have occurred prior to infection of the sampled tick, signals of positive selection likely reflect past selective pressures on the *Bb* genome.

### Do the same genes show signs of positive selection across ticks?

We compared patterns of within-tick *Bb* variation across multiple ticks and found that positively selected genes were strongly associated across pairs of ticks (Fig 4B). While ticks samples in this study are not independent samples due to the common ancestry of *Bb* across ticks, comparing the genes with signals of positive selection across ticks allows us to identify genes with the strongest signals of historic positive selection. The genes most commonly experiencing positive selection included several adhesins exposed on the bacterial surface including decorin-binding proteins A and B (*dbpA* and *dbpB*), which enable *Bb* dissemination in vertebrate hosts, and complement regulator-acquiring surface protein-1 (*CspA*), which plays a role in evasion of the vertebrate immune response by downregulating the alternate complement cascade (S3 Table)[31]. These surface-expressed bacterial genes including known immunogens are likely experiencing balancing selection imposed by vertebrate host immune responses. Further, a functional cluster involved with rRNA/ribosomal binding and another for zinc ion binding were enriched in the genes experiencing positive selection[32]. Each tick comprises a sample of the *Bb* diversity circulating in the enzootic cycle; genes with a consistent signal of positive selection across ticks are likely to drive *Bb* diversification.

We additionally examined evidence of more recent selection, occurring within ticks classified as singly infected. In the 30 ticks classified as singly infected, only two genes held a signal of positive selection (dN/dS > 1), reflecting both the low levels of within-tick *Bb* variation and strong purifying selection in singly infected ticks. The complement regulator-acquiring surface protein-1 (*CspA*) was under positive selection in 17% (5/30) of singly infected ticks and cysteine desulfarase (*csd*, probable annotation status) showed a signal of positive selection in one singly infected tick. The finding that *CspA* harbors a signal of positive selection both in multiple and single strain *Bb* infections suggests strong selective pressure imposed by the vertebrate immune system capable of imposing strong selection even on relatively clonal *Bb* populations in ticks. Again, even for the ticks classified as “singly infected,” selection on the sampled *Bb* population likely did not occur within the tick vector but within the vertebrate host.

Here, we present preliminary evidence of positive selection shaping *Bb* evolutionary history. However, positive selection may be difficult to detect within populations [33] and may occur at a scale finer than the whole gene (i.e. short functional fragments of the genes may be experiencing positive selection).

### Tick vectors of *B*. *burgdorferi* offer a simplified model for study of within-host ecological and evolutionary processes

Here, we develop a sensitive and specific method to identify within-tick *Bb* variants and find that within-tick *Bb* diversity is much higher than previously described[34,35]. The majority of ticks harbor levels of within-tick diversity consistent with a mixed infection, indicating that *Bb* is maintained in natural transmission cycles as multiple clonal cohorts. Further, we find preliminary evidence of within-tick *Bb* competition and detect signatures of population-level diversifying selection within multiply infected ticks and across ticks.

Characterizing circulating *Bb* diversity is of significant evolutionary interest because of the debate over the mechanisms maintaining extensive sympatric *Bb* diversity [16,36,37]. Here, we examine a cross-sectional snapshot of within-tick *Bb* diversity; further study of within-tick and host *Bb* diversity over a tick-vertebrate transmission cycle will help identify selective and neutral processes shaping *Bb* diversity, i.e.[8,38]. Modeling studies are needed to test hypotheses about how within-tick and within-host ecological processes drive *Bb* evolution.

Patterns of *Bb* diversity are of major epidemiological interest because *Bb* strains vary in virulence[39–42]. Several *Bb* genes with a signature of positive selection identified this study include those important for dissemination and establishment of infection in vertebrates. Several of these genes are similarly involved in dissemination and adhesion in human hosts, suggesting that that *Bb* virulence in humans may continue to evolve. We demonstrate that nymphal ticks frequently acquire diverse *Bb* populations; however, it remains unknown how much *Bb* diversity is transmitted to humans (i.e. if a transmission bottleneck exists). Characterizing human *Bb* infections with population deep sequencing will help us understand the prevalence of mixed infections in humans and measure virulence in single and mixed strain *Bb* infections. Complex interactions between coinfecting strains and the host may result in mixed infections with a range of virulence outcomes [6]; predicting overall virulence of mixed *Bb* infections in humans requires empirical research and modeling approaches.

By focusing on tick-borne *Bb*, we demonstrate that individual vectors can serve as epidemiological sentinels. Pathogen variation within infected disease vectors may reveal important epidemiological information such as the magnitude of pathogen diversity present in a single infectious bite (strain diversity in potential exposures) and the virulence/resistance genes undergoing population-level selection. This information is useful for predicting entomological risk. For example, surveys of within-vector diversity may identify areas at risk of frequent transmission of mixed infections that may be difficult to identify with traditional pathogen genotyping methods [13]. Further, characterizing variation and/or signatures of selection on pathogen drug resistance genes could help inform predictions about spread of drug resistance and identify areas at heightened risk for transmission of drug-resistant clones which may often be difficult to detect in mixed human infections [43]. Human disease vectors including mosquitoes and ticks transmit of all circulating pathogen genomic variation and sampling is non-invasive compared to sampling human/vertebrate hosts[44]. Investigating within-vector pathogen diversity can be a useful and unbiased way to survey circulating pathogen diversity and identify the evolutionary and ecological drivers shaping pathogen diversity.

## Materials and Methods

### Sample collection

We collected 98 *Bb*-infected nymphal *Ixodes scapularis* ticks from the widest available spatial and temporal range (i.e. including ticks sampled from 1984–2013) (S1 Fig). Tick collections, DNA extractions, and qPCR testing for *Bb* infection followed described protocols[25].

### Short-read sequencing

Genomic libraries were prepared from infected tick samples. *Bb* DNA was captured using a custom hybridization capture array method[25]. Sequencing with 75-bp paired end reads was conducted on an Illumina HiSeq 2500 at the Yale Center for Genomic Analysis. We included samples with average *Bb* coverage > 40X in all analyses. Using a more stringent coverage threshold of 100X, 72.1% (49/68) ticks are estimated to harbor mixed infections compared to 69.4% (68/98) in samples with 40X coverage. Short-read sequence data were submitted to the NCBI Short Read Archive (SRA; http://www.ncbi.nlm.nih.gov/sra/), SRA accession: SRP058536.

### Within-tick variant identification

We focused analysis on single nucleotide polymorphisms (SNPs) on the *Bb* linear chromosome (910724-bp) and the two best-characterized and most conserved plasmids, lp54 (53657-bp) and cp26 (26498-bp) because plasmid content is highly variable across *Bb* strains[45,46]. This captures 65% of the total *B31* reference genome.

We generated a consensus sequence for each within-tick *Bb* population and performed all mapping and variant calling with respect to the ticks’ consensus *Bb* sequence, maximizing sensitivity. (We did not conduct *de novo* assembly of consensus sequences because we use mixed DNA samples. After hybrid capture of *Bb*, ~40% of our sequence reads are not *Bb*-derived and likely constitute tick and other environmental DNA.) First, raw sequence reads for each sample were aligned to the *Bb* reference genome strain B31 [47,48] using BWA mem (v. 0.7.7) [49]. Duplicate sequence reads were marked and excluded from downstream analysis, using the Picard Suite (v. 1.117) MarkDuplicates (http://picard.sourceforge.net). Variants with respect to strain B31[47,48] were identified with GATK HaplotypeCaller (ploidy set to 1) and tick-specific consensus sequences were reconstructed with GATK FastaAlternateReferenceMaker[50]. Next, we remapped raw reads to the tick-specific consensus sequence with BWA mem (v. 0.7.7) [49], removed duplicates with Picard Suite (v. 1.117) MarkDuplicates (http://picard.sourceforge.net), and realigned reads around potential indels with GATK IndelRealigner[50].

We generated pileup files with SAMTOOLS v. 1.1 mpileup[51] and identified within-tick *Bb* polymorphisms using a set of sequence and mapping quality thresholds which allowed us to distinguish true within-tick *Bb* variants from noise due to sequencing and mapping error.

We restricted variant calling to iSNVs with an allele frequency > 3%. (Minor alleles at lower frequencies were difficult to distinguish from sequencing error.) We included only sites with coverage >10X, including > 5X coverage on the forward and reverse strands and > 2X coverage of each allele on each strand. (Filters out low coverage sites and strand bias, a type of sequencing error in which genotypes inferred by forward and reverse strands conflict.) We included only sites with Phred-scaled base quality > 30 for major and minor allele calls (filters out potential sequencing error); Phred-scaled mapping quality > 30 for major and minor allele calls (filters for mapping error); and Phred-scaled mapping quality difference between reads supporting major and minor allele < 3 (filters out iSNVs if mapping quality is significantly higher for major allele, eliminates false positive iSNVs called in the repetitive right-end of the *Bb* chromosome.).

We included only iSNVs with an average base position of the reads supporting the major and minor allele between 5 and 70 (75-bp reads). (Filters out sequencing errors potentially introduced if low quality bases are called at the beginning and ends of sequence reads.) We included only iSNVs where the percentage indels called at position < 20%. (Filters out iSNV genotype calling errors introduced at sites of putative indels.)

We included only sites passing the Strand Bias Fisher’s exact test (null hypothesis: allele call is not associated with strand), p-value > 10^−5^. (Filters out strand bias, a type of sequencing error in which genotypes inferred by forward and reverse strands conflict.) We included only sites passing the following Rank Sum Tests (null hypothesis: reads covering major and minor alleles are not associated with strand): Base Quality Rank Sum Test p-value > 10^−5^, Mapping Quality Rank Sum Test p-value > 10^−5^, and Tail Distance Rank Sum Test p-value > 10^−5^. (Rank sum tests harness differences in quality between major and minor alleles to filter out false positive iSNVs).

Thresholds were chosen in order to first maximize specificity (i.e. reduce probability of identifying false iSNVs) and secondly to maximize sensitivity (i.e. increase probability of identifying true iSNVs). Thresholds were based on those developed by Lieberman et al.[10] and updated to maximize specificity and sensitivity of variant calling for simulated *Bb* genomic mixtures.

### Threshold for *in situ* evolution

We summarized the level of within-tick *Bb* diversity for each tick by calculating genetic distance, defined as the sum of minor allele frequencies of each tick’s *Bb* population[10,38,52]. Genetic distance *d*_*i*_ for tick *i* is the sum of minor allele frequencies *p* over each callable site (site with > 40X coverage) *n* along the *Bb* genome: $d_{i}=\sum_{n}p_{n}$.

Given an estimated duration of tick infection [18,19] and range of estimates of *Bb* mutation rate[53], we estimate the maximum *d* consistent with *in situ* evolution: *d* = *ut* where *u* is the estimated *Bb* mutation rate and *t* is the duration of tick infection.

The duration of tick infection is the time from the larval bloodmeal to the nymphal bloodmeal: ~340–375 days (Fig 1, S4 Fig). Tick seasonality varies spatially (specifically, seasonality is known to differ between the upper Midwest and Northeast)[54] and is predicted to shift in response to climate change [55]; therefore, we considered a biologically realistic range of estimates of duration of infection [19,56]. In the Northeast United States, where extensive field data on tick seasonality is available, larval ticks feed in two cohorts: in early spring and in late summer. For larval ticks feeding in the early cohort (similar to seasonality observed in the upper Midwest), mean duration of tick infection is 376 days, for larval ticks feeding in the later cohort, mean duration of infection is 340 days [19,56,57] (S4 Fig). Note that duration of infection estimates represent an upper bound as ticks were sampled *before* their nymphal bloodmeal. We found that genetic distance is insensitive to estimated duration of infection, we therefore conservatively use the maximum duration of infection *t*_max_ of 376 days for threshold determination.

Though estimates of *Bb* within-tick generation time and mutation rate are unavailable[53], short term mutation rate estimates for other bacterial genomes are on the order of ~1 x 10^−7^ mutations/site/year [58–64], yielding an estimated *d* of 0.10 mutations consistent with *in situ* evolution. Conservatively assuming a mutation rate of 1 x 10^−6^ mutations/site/year, the within-host evolutionary rate of fast-evolving bacteria like *Staphylococcus aureus* and *Streptococcus pneumoniae*[58,63,65,66], the maximum genetic distance possibly attributable to *in situ* evolution, *d*_max_ increases by one degree of magnitude to 1.02 mutations.

### Threshold for sequencing/mapping error

To establish a threshold for distinguishing true minority variants from background sequencing or mapping error, we generated Illumina sequence data including an Illumina error profile *in silico* for three diverse *Bb* reference genomes, B31, N40, JD1, [47,48] (pairwise divergence, 4.60–6.01%) with the ART read simulator[67]. We generated ten independent sequence data sets for each reference genome at 100X coverage. We then identified iSNVs and calculated *Bb* genetic distance, as described above (S2 Fig). As each read-set represents an isogenic *Bb* population (the reads are simulated from a single reference genome), any iSNVs identified are false positives. The maximum genetic distance (2.33 mutations) is the threshold above which it is possible to distinguish “true” biological variants from sequencing or mapping errors (S2 Fig). A genetic distance greater 3.15, the sum of *d*_max_ due to *in situ* evolution (1.03 mutations) and the conservative sequencing/mapping error threshold (2.33 mutations) constitutes evidence of a mixed infection.

### Selection detection

We examined the distribution of within-tick polymorphisms for each of the 876 *Bb* genes annotated in the ENA gene build (genome-version GCA_000008685.2). Here, we are interested in the biological consequence of mutations away from the consensus allele (our best estimate of the allele present in the majority *Bb* strain infecting each tick) to observed minor alleles. For a single strain infection, this allows us to assess the biological consequence of mutation away from the single strain. For a mixed strain infection, this allows us to assess the biological consequence of mutations between the two (or more) circulating *Bb* strains infecting the tick. To predict the biological consequence of each iSNV, we generated VCF files with VarScan[68], extracted iSNVs, and used SnpEff [30] to predict variant effect. Because we do not have haplotype information, we determined dN/dS ratios for each codon within each within-tick *Bb* population with the counting method[69]. We counted the number of synonymous and non-synonymous iSNVs in each codon, applied a correction for multiple substitutions at a site[70], and normalized counts using the non-synonymous and synonymous sites available for mutation determined in HyPhy[71].

We compared the list of positively selected *Bb* genes (dN/dS > 1) within each pair of ticks and tested association between sets of positively selected genes with Fisher’s exact tests implemented in the R package GeneOverlap[72]. We examined the functional annotation of genes undergoing positive selection as compared to the background gene set comprised of all *Bb* genes with DAVID[32].

## Supporting Information

S1 Fig Sampling locations. Site of *Ixodes scapularis* collection (a) colored by sampling year (b). Sampling locations are jittered for visibility.
(TIF)

S2 Fig Threshold for minority variant calling. To establish a threshold for distinguishing true minority variants from background sequencing and mapping error (false positive polymorphisms), we simulated paired-end reads generated from isogenic populations with three different reference genomes (B31, JD1, and N40) with an empirically derived Illumina sequencing error profile. We followed the protocol described in the Methods: we mapped simulated reads to the B31 reference genome and called iSNVs. Boxplots of (a) the number of iSNVs and (b) measured genetic distance *d* for these simulated isogenic populations represents the threshold above which it is possible to distinguish biological variation from noise generated by sequencing and mapping errors. Bold lines indicate the median value, the boxes span the interquartile range and whiskers extend to the extremes of the sampled values, excluding outliers. We conservatively use the maximum *d* (2.33 mutations) estimated in simulated isogenic populations (in simulated JD1 samples), as a threshold for the *d* possibly attributable to mapping/sequencing errors.
(TIFF)

S3 Fig Sensitivity and specificity of minority variant identification. We simulated ten *in silico* mixed infections for which the minority variant comprised 1–50% of the total within-host *Bb* population and identified iSNVs. For each proportion of minority variant, sensitivity (true positive rate), the proportion of positives (true SNP sites between the two mixed genomes) correctly identified by our minority variant caller (a) and specificity (true negative rate), the proportion of negatives (conserved sites) correctly identified as such (b).
(TIFF)

S4 Fig *Ixodes scapularis* seasonal feeding activity. *I*. *scapularis* host-seeking (feeding) activity estimated with larval and nymphal tick burdens on each trapped *Peromyscus leucopus* (white-footed mouse) host shown for a two-year period. Larval tick activity (dashed blue line) is followed by molting and winter diapause in the first year. Nymphal tick activity (solid red line) is shown for the second year. Larval ticks in the Northeast feed in two cohorts: in spring and late summer. Green lines depict the duration of *Bb* infection for a single tick acquiring *Bb* as a larval tick, molting, and host-seeking again as a nymphal tick. The dotted green line represents a longer duration of *Bb* infection for larval ticks in the early cohort (~376 days) and the solid green line represents the shorter duration of *Bb* infection for larval ticks feeding in the late cohort (~340 days). Parameter estimates are derived from field data collected on Block Island, Rhode Island[56]. The functional forms of tick burden curves are previously described[19,56].
(TIF)

S5 Fig *Bb* burden in singly infected and multiply infected hosts. Each point represents the number of *Bb* genome equivalents (measured by qPCR) and red bars indicate the median. There is no significant difference between the *Bb* burden and infection status (Mann-Whitney test, p = 0.543).
(TIFF)

S6 Fig Within-tick *Bb* diversity across sampling years. Within-tick *Bb* genetic distance for all samples (a) and for the 68 samples collected in the Northeast (b). Within-tick *Bb* genetic distance is not associated with sampling year for all samples (F-test, p = 0.47) nor for northeastern samples (F-test, p = 0.54).
(TIFF)

S7 Fig Within-tick *Bb* diversity across sampling regions. Within-tick *Bb* genetic distance (log-scale) for samples collected in Canada, the Midwest, Northeast, and South (Virginia). Samples collected in Virginia had significantly lower within-tick *Bb* diversity than samples from any other region (Wilcoxon rank sum tests, regional comparisons of Virginia vs. Midwest, Canada, and Northeast, all p < 0.05).
(TIFF)

S8 Fig The distribution of iSNVs across the plasmids and two known antigens in a representative multiply infected host (Bbcap17). The iSNV frequencies across plasmids lp54 (a) and cp26 (b) holds a signature of two minority variant strains. iSNVs are distributed across each plasmid (red bars indicate the location of the antigens *dbpA* and *ospC* respectively). The distribution of iSNVs in *dbpA* (c) and *ospC* (d) demonstrates that iSNVs are localized to specific areas of each antigen, identifying regions for focused functional exploration.
(TIF)

S1 Table Nymphal *I*. *scapularis* samples. Sample name, sampling site, state, collection year, q-PCR determined *Bb* copy number, mean chromosomal coverage, within-host genetic distance (*d*), and number of iSNVs identified.
(DOC)

S2 Table Evidence of purifying selection across the *Bb* chromosome and plasmids. The number of synonymous and non-synonymous sites, the average number of non-synonymous iSNVs (dN) and synonymous iSNVs), and the dN/dS ratio (corrected for multiple substitutions at the same site). Standard deviations are in parentheses.
(DOC)

S3 Table *Bb* genes under positive selection. (Top 50 most commonly repeated across hosts.)
(DOC)

S1 Text Intrahost variant identification. (DOCX)
