# Supplementary figures and images for: Vectors as Epidemiological Sentinels: Patterns of Within-Tick Borrelia burgdorferi Diversity
Source: PLoS Pathog. 2016 Jul 14;12(7):e1005759. doi: 10.1371/journal.ppat.1005759 (PMC4944968; doi:10.1371/journal.ppat.1005759)

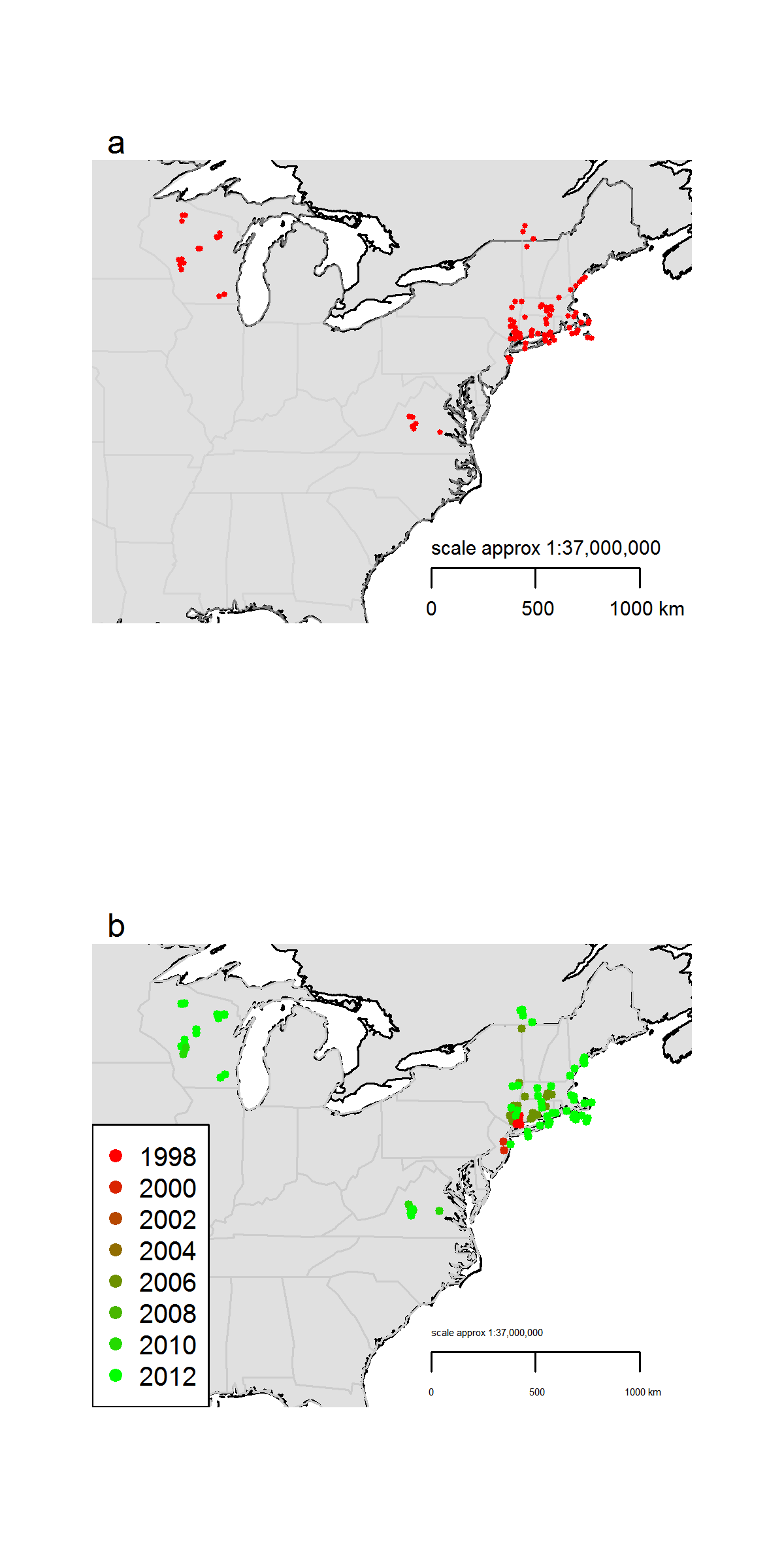

Supplement: S1 Fig — Site of Ixodes scapularis collection (a) colored by sampling year (b). Sampling locations are jittered for visibility. (TIF) [file ppat.1005759.s001.tif]

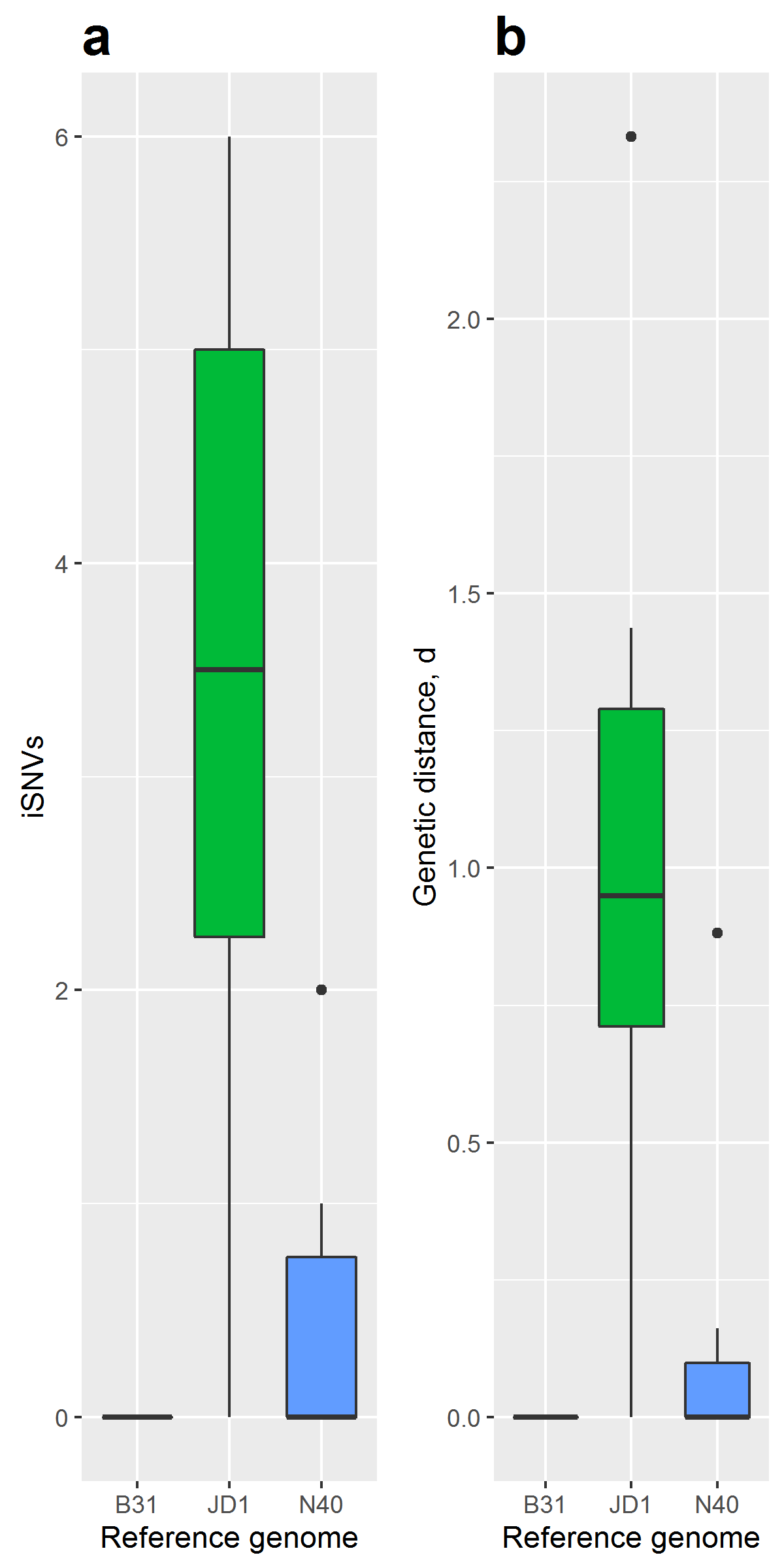

Supplement: S2 Fig — To establish a threshold for distinguishing true minority variants from background sequencing and mapping error (false positive polymorphisms), we simulated paired-end reads generated from isogenic populations with three different reference genomes (B31, JD1, and N40) with an empirically derived Illumina sequencing error profile. We followed the protocol described in the Methods: we mapped simulated reads to the B31 reference genome and called iSNVs. Boxplots of (a) the number of iSNVs and (b) measured genetic distance d for these simulated isogenic populations represents the threshold above which it is possible to distinguish biological variation from noise generated by sequencing and mapping errors. Bold lines indicate the median value, the boxes span the interquartile range and whiskers extend to the extremes of the sampled values, excluding outliers. We conservatively use the maximum d (2.33 mutations) estimated in simulated isogenic populations (in simulated JD1 samples), as a threshold for the d possibly attributable to mapping/sequencing errors. (TIFF) [file ppat.1005759.s002.tiff]

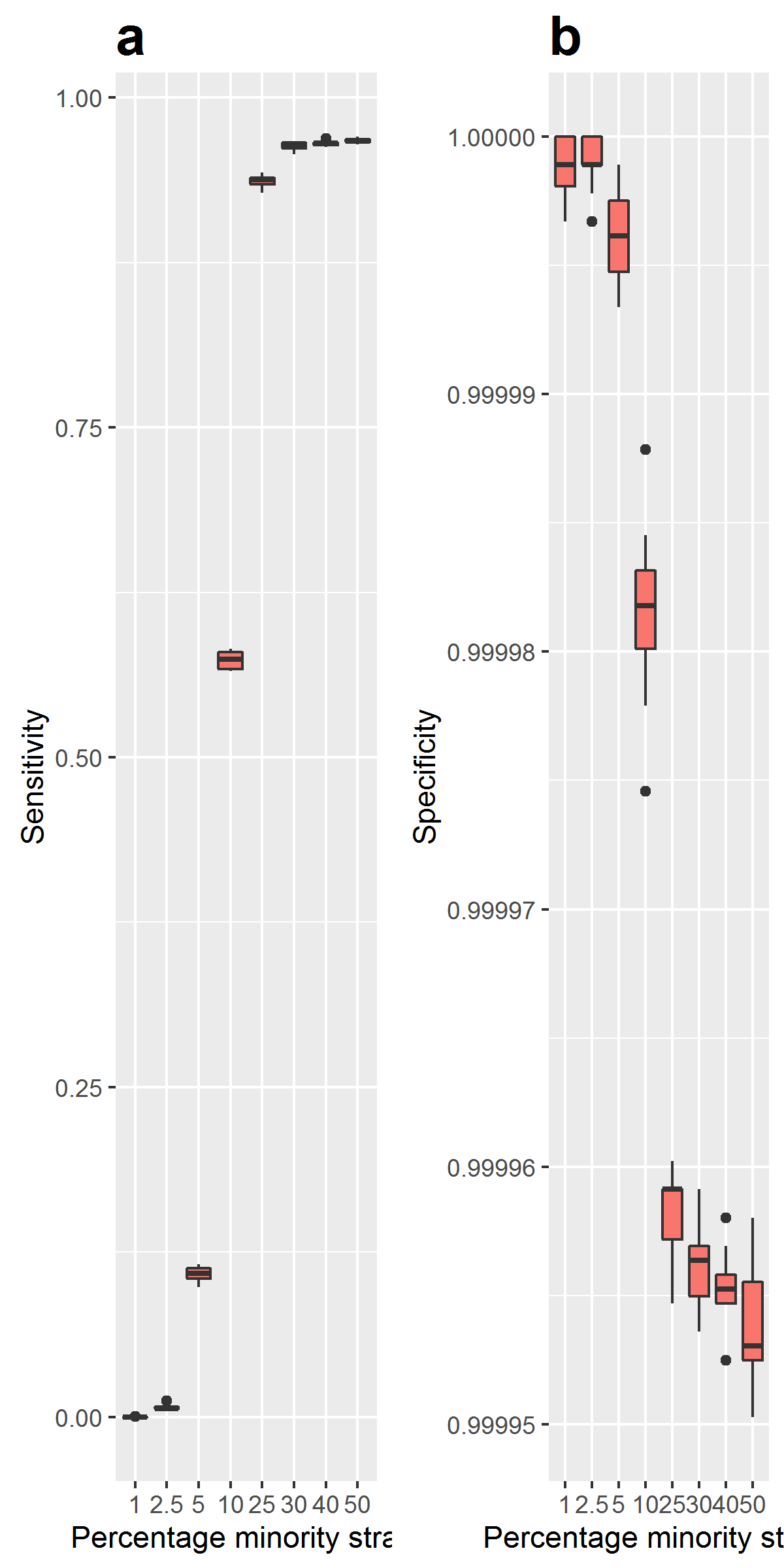

Supplement: S3 Fig — We simulated ten in silico mixed infections for which the minority variant comprised 1–50% of the total within-host Bb population and identified iSNVs. For each proportion of minority variant, sensitivity (true positive rate), the proportion of positives (true SNP sites between the two mixed genomes) correctly identified by our minority variant caller (a) and specificity (true negative rate), the proportion of negatives (conserved sites) correctly identified as such (b). (TIFF) [file ppat.1005759.s003.tiff]

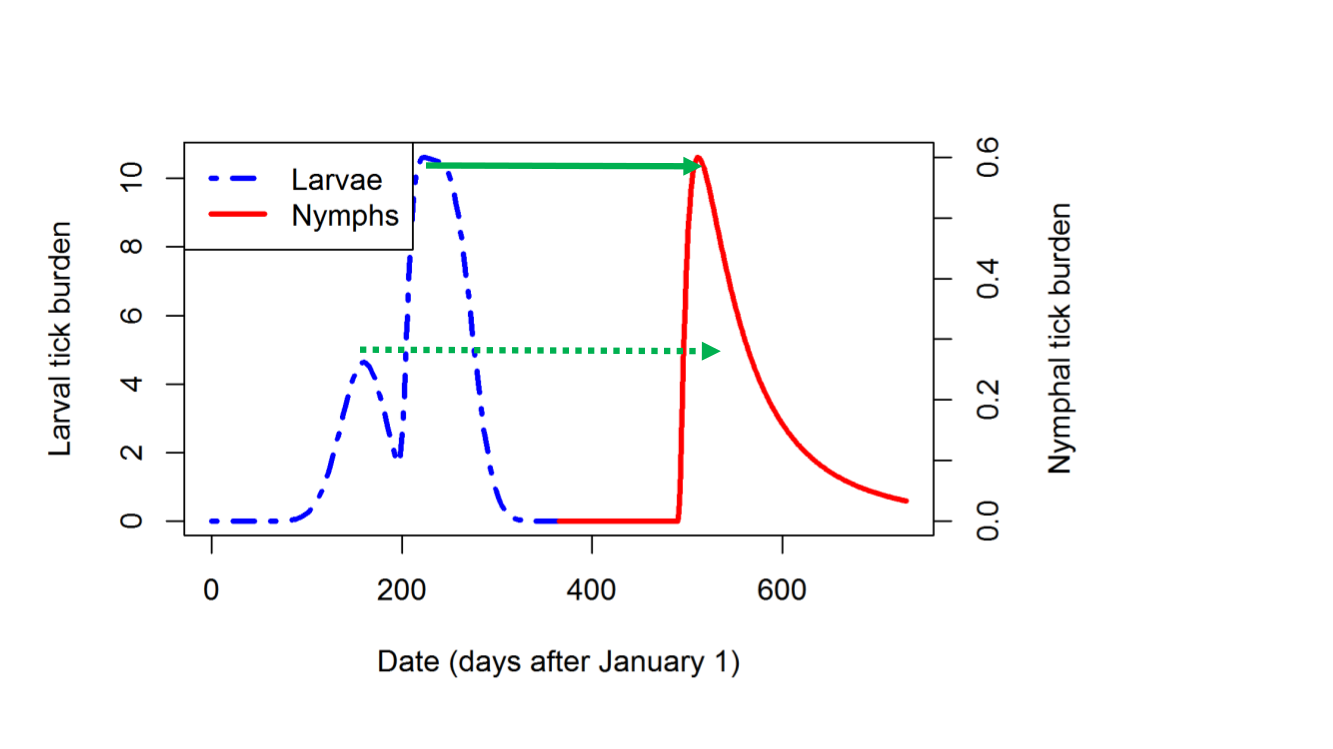

Supplement: S4 Fig — I. scapularis host-seeking (feeding) activity estimated with larval and nymphal tick burdens on each trapped Peromyscus leucopus (white-footed mouse) host shown for a two-year period. Larval tick activity (dashed blue line) is followed by molting and winter diapause in the first year. Nymphal tick activity (solid red line) is shown for the second year. Larval ticks in the Northeast feed in two cohorts: in spring and late summer. Green lines depict the duration of Bb infection for a single tick acquiring Bb as a larval tick, molting, and host-seeking again as a nymphal tick. The dotted green line represents a longer duration of Bb infection for larval ticks in the early cohort (~376 days) and the solid green line represents the shorter duration of Bb infection for larval ticks feeding in the late cohort (~340 days). Parameter estimates are derived from field data collected on Block Island, Rhode Island[56]. The functional forms of tick burden curves are previously described[19,56]. (TIF) [file ppat.1005759.s004.tif]

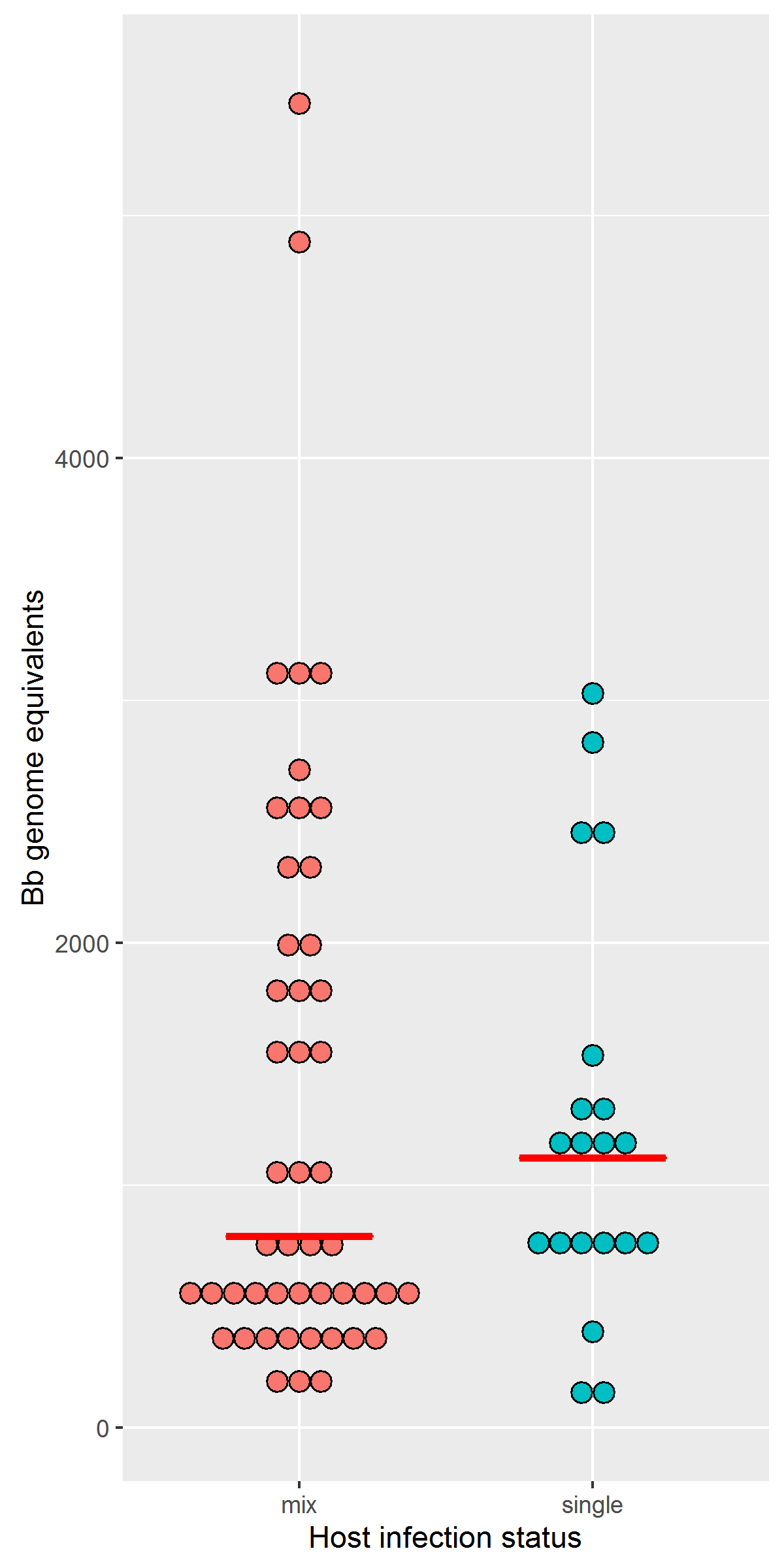

Supplement: S5 Fig — Each point represents the number of Bb genome equivalents (measured by qPCR) and red bars indicate the median. There is no significant difference between the Bb burden and infection status (Mann-Whitney test, p = 0.543). (TIFF) [file ppat.1005759.s005.tiff]

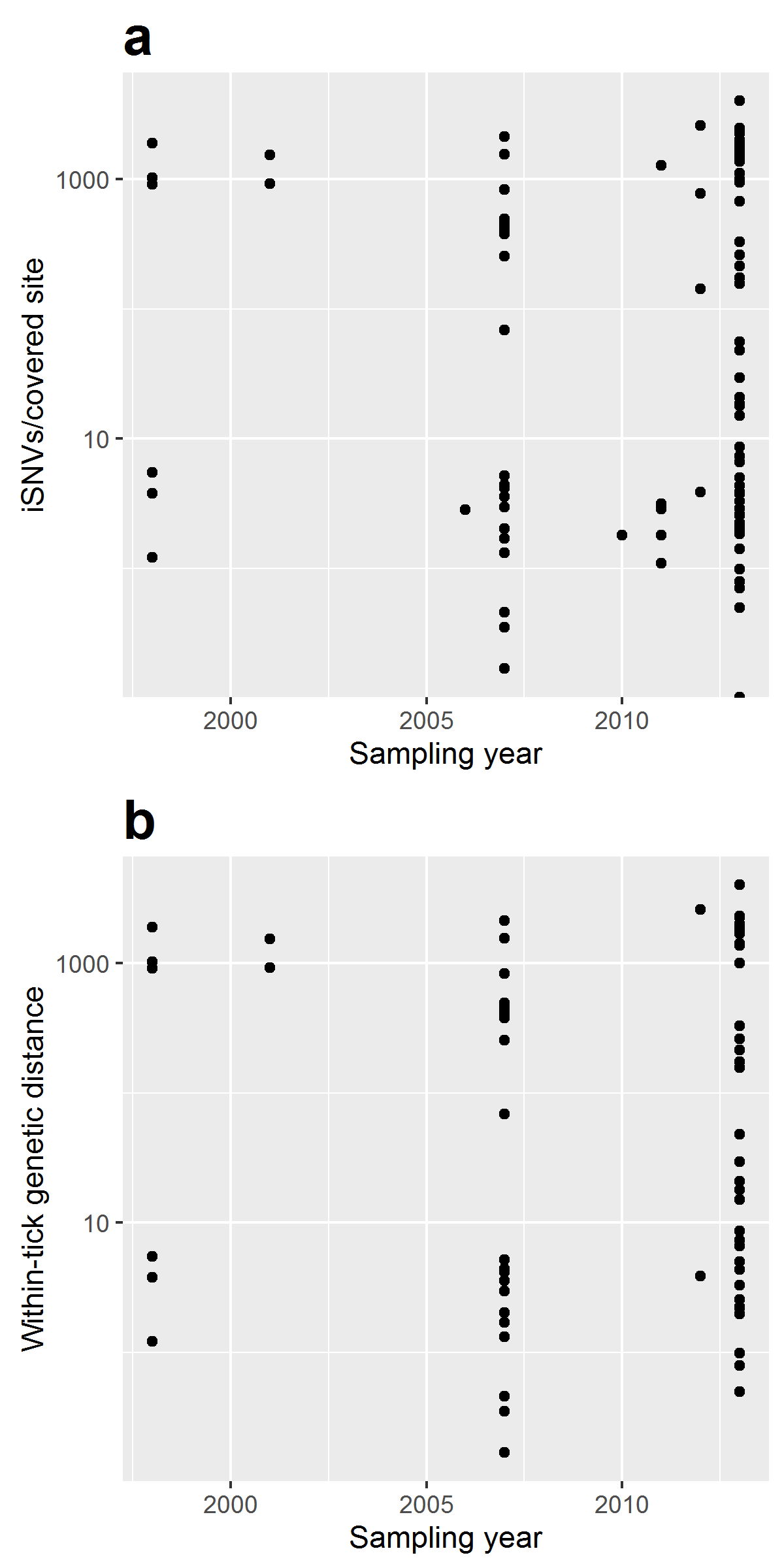

Supplement: S6 Fig — Within-tick Bb genetic distance for all samples (a) and for the 68 samples collected in the Northeast (b). Within-tick Bb genetic distance is not associated with sampling year for all samples (F-test, p = 0.47) nor for northeastern samples (F-test, p = 0.54). (TIFF) [file ppat.1005759.s006.tiff]

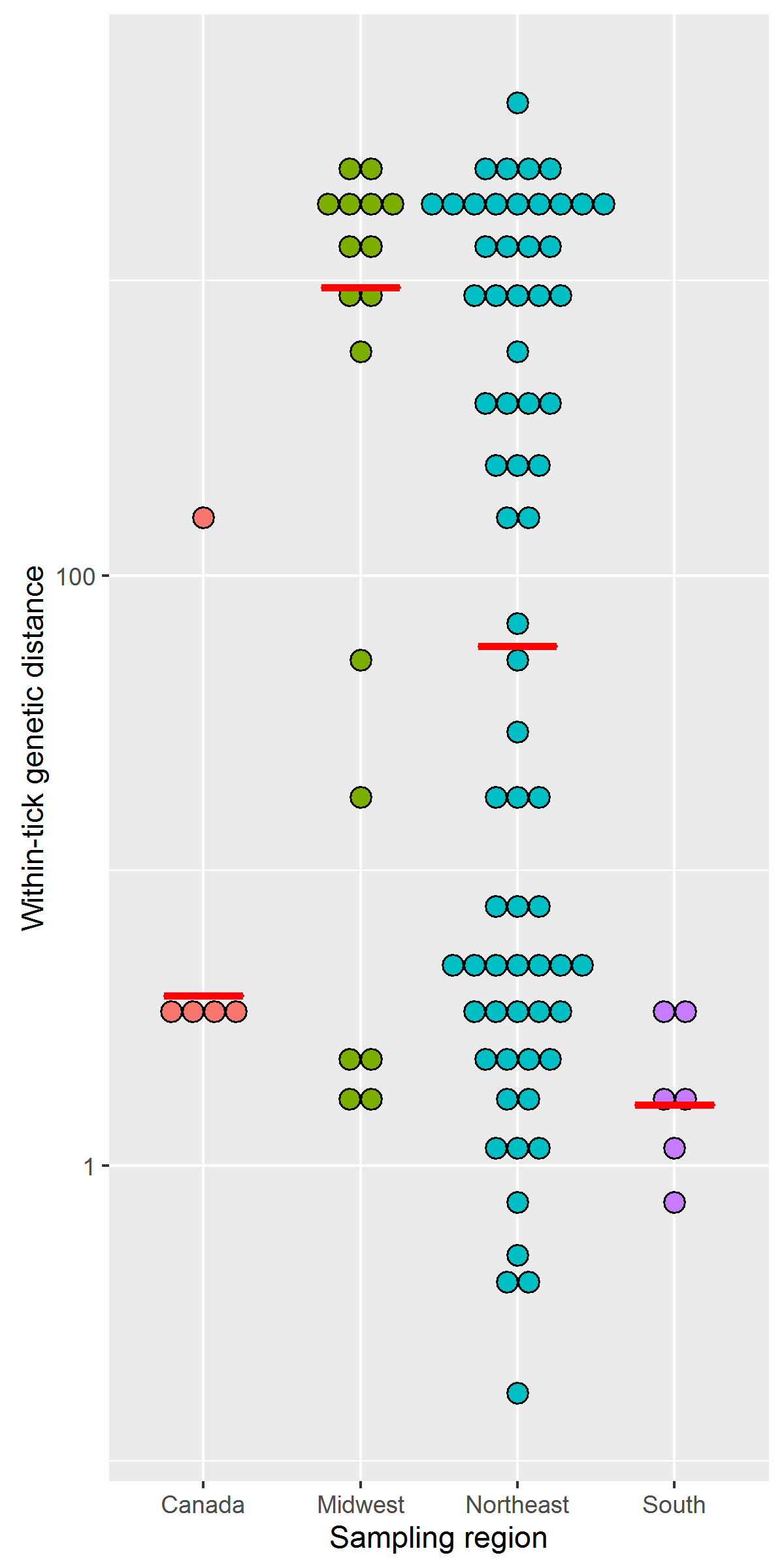

Supplement: S7 Fig — Within-tick Bb genetic distance (log-scale) for samples collected in Canada, the Midwest, Northeast, and South (Virginia). Samples collected in Virginia had significantly lower within-tick Bb diversity than samples from any other region (Wilcoxon rank sum tests, regional comparisons of Virginia vs. Midwest, Canada, and Northeast, all p < 0.05). (TIFF) [file ppat.1005759.s007.tiff]

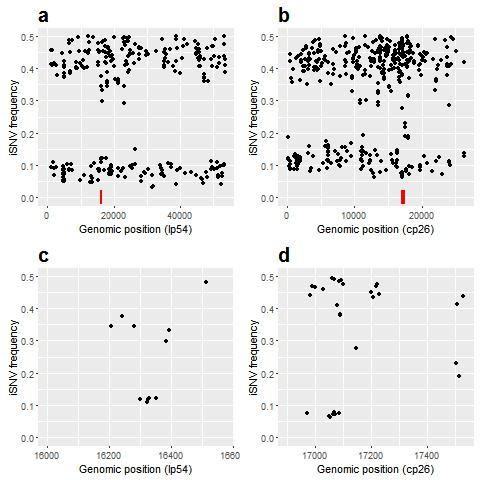

Supplement: S8 Fig — The iSNV frequencies across plasmids lp54 (a) and cp26 (b) holds a signature of two minority variant strains. iSNVs are distributed across each plasmid (red bars indicate the location of the antigens dbpA and ospC respectively). The distribution of iSNVs in dbpA (c) and ospC (d) demonstrates that iSNVs are localized to specific areas of each antigen, identifying regions for focused functional exploration. (TIF) [file ppat.1005759.s008.tif]
