# Supplementary material for: Vectors as Epidemiological Sentinels: Patterns of Within-Tick Borrelia burgdorferi Diversity
Source: PLoS Pathog. 2016 Jul 14;12(7):e1005759. doi: 10.1371/journal.ppat.1005759 (PMC4944968; doi:10.1371/journal.ppat.1005759)
Supplement: S1 Table — Sample name, sampling site, state, collection year, q-PCR determined Bb copy number, mean chromosomal coverage, within-host genetic distance (d), and number of iSNVs identified. (DOC) [file ppat.1005759.s009.doc]

**S1 Table. Nymphal *I. scapularis* samples.** Sample name, sampling site, state, collection year, q-PCR determined *Bb* copy number, mean chromosomal coverage, within-host genetic distance ($d$), and number of iSNVs identified.

| **Sample** | **Site** | **State** | **Collection Year** | ***Bb* Copy Number** | **Mean Coverage** | $d$***d*** | **iSNVs** |
| --- | --- | --- | --- | --- | --- | --- | --- |
| Bbcap10 | James Baird State Park | NY | 2007 | 2828 | 307 | 1 | 13 |
| Bbcap12 | James Baird State Park | NY | 2007 | 1631 | 104 | 376 | 2957 |
| Bbcap13 | James Baird State Park | NY | 2007 | 3030 | 334 | 2 | 16 |
| Bbcap14 | James Baird State Park | NY | 2007 | 480 | 78 | 4 | 17 |
| Bbcap16 | Yale Meyers Forest | CT | 2007 | 1086 | 72 | 3 | 10 |
| Bbcap17 | Grand Isle State Park | VT | 2007 | 2476 | 221 | 2102 | 6818 |
| Bbcap2 | FDR State Park | NY | 2007 | 3156 | 127 | 826 | 4391 |
| Bbcap20 | Grafton Lakes State Park | NY | 2007 | 783 | 73 | 449 | 2695 |
| Bbcap21 | Moorse State Park | MA | 2007 | 787 | 73 | 2 | 7 |
| Bbcap22 | Moorse State Park | MA | 2007 | 2387 | 600 | 0 | 4 |
| Bbcap23 | Moorse State Park | MA | 2007 | 582 | 78 | 4 | 17 |
| Bbcap24 | Moorse State Park | MA | 2007 | 301 | 43 | 254 | 1242 |
| Bbcap28 | Mount Riga State Park | CT | 2007 | 701 | 73 | 488 | 2699 |
| Bbcap29 | Lake Gaillard | CT | 2007 | 2283 | 264 | 2112 | 6826 |
| Bbcap3 | FDR State Park | NY | 2007 | 388 | 53 | 69 | 441 |
| Bbcap30 | Lake Gaillard | CT | 2007 | 3099 | 625 | 4 | 32 |
| Bbcap31 | Lake Gaillard | CT | 2007 | 1800 | 310 | 1549 | 5873 |
| Bbcap32 | Lake Gaillard | CT | 2007 | 813 | 87 | 0 | 3 |
| Bbcap4 | FDR State Park | NY | 2007 | 603 | 147 | 5 | 38 |
| Bbcap5 | FDR State Park | NY | 2007 | 1472 | 110 | 409 | 3249 |
| Bbcap6 | FDR State Park | NY | 2007 | 2641 | 318 | 4 | 22 |
| Bbcap9 | FDR State Park | NY | 2007 | 1266 | 134 | 0 | 3 |
| BEP-10 | Big Eau Pleine County Park | WI | 2013 | NA | 292 | 2452 | 7347 |
| BEP-6 | Big Eau Pleine County Park | WI | 2013 | NA | 97 | 18 | 211 |
| BRF-226 | Black River Falls State Forest | WI | 2013 | NA | 393 | 669 | 4442 |
| BRF-231 | Black River Falls State Forest | WI | 2013 | NA | 626 | 56 | 1400 |
| BRF-236 | Black River Falls State Forest | WI | 2013 | NA | 53 | 3 | 9 |
| CT-1807 | Barn Island WMA | CT | 2013 | 1870 | 267 | 2241 | 4926 |
| CT-1827 | Barn Island WMA | CT | 2013 | 567 | 75 | 1992 | 4569 |
| CT-2408 | 50-Foot Cliff | CT | 2013 | 537 | 145 | 29 | 405 |
| CT-2422 | 50-Foot Cliff | CT | 2013 | 970 | 219 | 21 | 68 |
| CT-2493 | Rocky Neck SP | CT | 2012 | NA | 194 | 2582 | 11259 |
| FB-143 | Flambeau River State Forest | WI | 2013 | NA | 790 | 2 | 18 |
| FB-153 | Flambeau River State Forest | WI | 2013 | NA | 243 | 1104 | 7599 |
| FB-158 | Flambeau River State Forest | WI | 2013 | NA | 47 | 1773 | 4116 |
| HIS-2939 | Maple Bacon | NY | 1998 | 619 | 249 | 1020 | 5267 |
| HIS-2944 | Maple Bacon | NY | 1998 | 282 | 110 | 6 | 15 |
| HIS-2946 | Maple Bacon | NY | 1998 | 715 | 348 | 1 | 10 |
| HIS-2951 | Tea Exp | NY | 1998 | 289 | 188 | 1871 | 5099 |
| HIS-2955 | Tea Exp | NY | 1998 | 194 | 110 | 907 | 2594 |
| HIS-2957 | Tea Exp | NY | 1998 | 632 | 109 | 4 | 13 |
| HIS-3040 | Naval Weapons Station Earle | NJ | 2001 | 545 | 52 | 1525 | 4219 |
| HIS-3047 | Naval Weapons Station Earle | NJ | 2001 | 2714 | 754 | 918 | 7438 |
| KM-102 | Kettle Moraine State Forest | WI | 2013 | NA | 989 | 1679 | 4848 |
| KM-115 | Kettle Moraine State Forest | WI | 2013 | NA | 43 | 1548 | 4639 |
| L06-CEM16 | Lunenburg | Nova Scotia | 2006 | 61 | 51 | 3 | 8 |
| MA-1670 | Manuel F. Correllus State Forest | MA | 2013 | 821 | 42 | 2 | 6 |
| MA-1720 | Squam Swamp | MA | 2013 | 2345 | 292 | 994 | 5424 |
| MA-1729 | Squam Swamp | MA | 2013 | 1160 | 107 | 2 | 13 |
| MA-1834 | Wompatuck State Park | MA | 2013 | 2001 | 504 | 214 | 4294 |
| MA-1839 | Wompatuck State Park | MA | 2013 | 396 | 199 | 7 | 33 |
| MA-1927 | Mount Holyoke Range State Park | MA | 2013 | 994 | 41 | 1680 | 3985 |
| MA-1928 | Mount Holyoke Range State Park | MA | 2013 | 451 | 104 | 9 | 37 |
| MA-2294 | Quashnet River State Park | MA | 2013 | 1142 | 43 | 1 | 1 |
| MA-2296 | Quashnet River State Park | MA | 2013 | NA | 418 | 1 | 14 |
| MA-2363 | Cuttyhunk | MA | 2013 | 479 | 106 | 15 | 46 |
| MA-2371 | Cuttyhunk | MA | 2013 | 325 | 72 | 3982 | 13448 |
| MA-2552 | Townsend State Forest | MA | 2013 | 1984 | 461 | 156 | 3378 |
| MA-2678 | Nickerson State Park | MA | 2013 | 2518 | 425 | 7 | 105 |
| MA-2682 | Nickerson State Park | MA | 2013 | 3070 | 383 | 2286 | 7085 |
| MB-44 | McCaslin Brook | WI | 2013 | NA | 123 | 2 | 7 |
| MB-48 | McCaslin Brook | WI | 2013 | NA | 772 | 1917 | 6852 |
| MB-63 | McCaslin Brook | WI | 2013 | NA | 346 | 943 | 7778 |
| MB-74 | McCaslin Brook | WI | 2013 | NA | 46 | 0 | 0 |
| ME-2735 | Cape Elizabeth | ME | 2013 | 1334 | 286 | 2 | 10 |
| ME-2737 | Cape Elizabeth | ME | 2013 | NA | 66 | 4 | 18 |
| ME-2739 | Cape Elizabeth | ME | 2013 | 1738 | 352 | 18 | 310 |
| MI-P4 | Nenominee County | MI | 2006 | NA | 853 | NA | NA |
| NH-2426 | Great Bay National Wildlife Refuge | NH | 2013 | 1538 | 422 | 2276 | 7304 |
| NH-2440 | Great Bay National Wildlife Refuge | NH | 2013 | NA | 87 | 1 | 8 |
| NJ-2805 | Naval Weapons Station Earle | NJ | 2013 | 797 | 108 | 1778 | 5696 |
| NY-1468 | Connetquot State Park | NY | 2013 | NA | 577 | 1984 | 8731 |
| NY-1478 | Connetquot State Park | NY | 2013 | 2525 | 608 | 3 | 20 |
| NY-1522 | Mashomack Preserve | NY | 2013 | 808 | 206 | 173 | 2640 |
| NY-1524 | Montauk Point State Park | NY | 2013 | NA | 73 | 48 | 274 |
| NY-2387 | Ward Pound Ridge | NY | 2013 | NA | 40 | 260 | 1028 |
| NY-2455 | James Baird State Park | NY | 2013 | 1141 | 51 | 328 | 1503 |
| NY-2464 | James Baird State Park | NY | 2013 | 486 | 105 | 1364 | 6350 |
| NY-2516 | Schodack Island State Park | NY | 2013 | 335 | 97 | 5 | 26 |
| NY-2523 | Shodack Island State Park | NY | 2013 | 5465 | 1083 | 1353 | 7510 |
| NY-2534 | Hither Hills State Park | NY | 2013 | 1538 | 428 | 3 | 11 |
| NY-2539 | Hither Hills State Park | NY | 2013 | 4893 | 1239 | 1904 | 4288 |
| NY-2600 | Hither Hills State Park | NY | 2013 | NA | 1000 | 1412 | 5392 |
| QC12-249 | Farnham | Quebec | 2012 | 356 | 650 | 142 | 3467 |
| QC13-332 | Mont-Saint Bruno | Quebec | 2013 | 576 | 215 | 4 | 17 |
| QC13-351 | Mont-Saint Bruno | Quebec | 2013 | 229 | 152 | 3 | 19 |
| QC13-6 | Mont-Saint Bruno | Quebec | 2013 | 103 | 40 | 4 | 11 |
| RI-855 | George Washington WMA | RI | 2012 | NA | 107 | 4 | 13 |
| T13-2047 | Ft. McCoy | WI | 2012 | NA | 122 | 775 | 4638 |
| T13-220 | Ft. McCoy | WI | 2011 | NA | 806 | 1267 | 4639 |
| T14-0159 | Ft. McCoy | WI | 2010 | NA | 1463 | 2 | 14 |
| VA-1942 | Lesesne State Forest | VA | 2011 | 764 | 184 | 3 | 15 |
| VA-2001 | Lesesne State Forest | VA | 2011 | 703 | 347 | 3 | 17 |
| VA-2058 | Goochland County | VA | 2011 | 397 | 46 | 1 | 3 |
| VA-2549 | Lesesne State Forest | VA | 2011 | 1295 | 243 | 2 | 16 |
| VA-5001 |  | VA | 2013 | NA | 59 | 1 | 4 |
| VA-5044 |  | VA | 2013 | NA | 246 | 1 | 4 |
| VA-5054 |  | VA | 2013 | NA | 3621 | 0 | 0 |
| WI-205 | Black River State Forest | WI | NA | NA | 142 | 2203 | 5981 |
