# Supplementary material for: Vectors as Epidemiological Sentinels: Patterns of Within-Tick Borrelia burgdorferi Diversity
Source: PLoS Pathog. 2016 Jul 14;12(7):e1005759. doi: 10.1371/journal.ppat.1005759 (PMC4944968; doi:10.1371/journal.ppat.1005759)
Supplement: S2 Table — The number of synonymous and non-synonymous sites, the average number of non-synonymous iSNVs (dN) and synonymous iSNVs), and the dN/dS ratio (corrected for multiple substitutions at the same site). Standard deviations are in parentheses. (DOC) [file ppat.1005759.s010.doc]

**S2 Table. Evidence of purifying selection across the *Bb* chromosome and plasmids.** The number of synonymous and non-synonymous sites, the average number of non-synonymous iSNVs (dN) and synonymous iSNVs), and the dN/dS ratio (corrected for multiple substitutions at the same site). Standard deviations are in parentheses.

| **Contig** | **Synonymous sites** | **Non-synonymous sites** | **dS** | | **dN** | | **dN/dS** | |
| --- | --- | --- | --- | --- | --- | --- | --- | --- |
| Chromosome | 223478.65 | 458489.19 | 2493.15 | (2334.02) | 618.29 | (694.74) | 0.12 | (0.10) |
| cp26 | 6177.35 | 13953.02 | 138.47 | (179.32) | 33.24 | (44.40) | 0.09 | (0.06) |
| lp54 | 10234.53 | 22151.05 | 82.79 | (76.08) | 38.11 | (40.81) | 0.26 | (0.32) |
