# Supplementary material for: Vectors as Epidemiological Sentinels: Patterns of Within-Tick Borrelia burgdorferi Diversity
Source: PLoS Pathog. 2016 Jul 14;12(7):e1005759. doi: 10.1371/journal.ppat.1005759 (PMC4944968; doi:10.1371/journal.ppat.1005759)
Supplement: S3 Table — (Top 50 most commonly repeated across hosts.) (DOC) [file ppat.1005759.s011.doc]

**S3 Table. *Bb* genes under positive selection***. (Top 50 most commonly repeated across hosts.)

| **Entry** | **Gene names** | **Status** | **Protein names**** | **Length** |
| --- | --- | --- | --- | --- |
| O50957 | cspA *BB*_A68 | unreviewed | **Complement regulator-acquiring surface protein 1 (CRASP-1)** | 251 |
| O51499 | *BB*_0549 | unreviewed | Uncharacterized protein | 132 |
| O51164 | *BB*_0139 | unreviewed | Uncharacterized protein | 103 |
| O51538 | *BB*_0592 | unreviewed | Caax amino protease family | 237 |
| O51300 | *BB*_0321 | unreviewed | Uncharacterized protein | 65 |
| O50917 | dbpA *BB*_A24 | reviewed | **Decorin-binding protein A** | 191 |
| H7C7K6 | *BB*_A59 | unreviewed | Uncharacterized protein | 79 |
| O50898 | *BB*_A04 | unreviewed | **S2 antigen** | 282 |
| P53363 | rsmG gidB *BB*_0177 | reviewed | Ribosomal RNA small subunit methyltransferase G | 208 |
| O51478 | *BB*_0528 | unreviewed | Aldose reductase, putative | 315 |
| O50982 | ch*Bb BB*_B06 | unreviewed | Chitibiose transporter protein ch*Bb* | 91 |
| H7C7P4 | *BB*_A64 | unreviewed | **P35 antigen** | 302 |
| O50946 | *BB*_A54 | unreviewed | Uncharacterized protein | 92 |
| G5IXH7 | *BB*_A0078 | unreviewed | Lipoprotein, putative | 74 |
| O51784 | *BB*_0844 | unreviewed | Lipoprotein, putative | 323 |
| O51484 | xth *BB*_0534 | unreviewed | Exodeoxyribonuclease III | 255 |
| O51447 | rplR *BB*_0494 | reviewed | 50S ribosomal protein L18 | 119 |
| O51411 | *BB*_0455 | unreviewed | DNA polymerase III, delta subunit superfamily | 329 |
| O50165 | *BB*_0378 | reviewed | Uncharacterized protein *BB*_0378 | 220 |
| Q44904 | fliZ *BB*_0276 | reviewed | Flagellar protein FliZ | 208 |
| O51190 | *BB*_0168 | unreviewed | DnaK suppressor, putative | 125 |
| O51163 | *BB*_0138 | unreviewed | Uncharacterized protein | 69 |
| O51146 | uppS *BB*_0120 | reviewed | Isoprenyl transferase | 230 |
| O51749 | tgt *BB*_0809 | reviewed | Queuine tRNA-ribosyltransferase | 375 |
| O51732 | *BB*_0792 | unreviewed | Uncharacterized protein | 207 |
| O51524 | *BB*_0577 | unreviewed | Uncharacterized protein | 195 |
| O51077 | *BB*_0048 | reviewed | Uncharacterized protein *BB*_0048 | 56 |
| O51061 | lepB *BB*_0030 | unreviewed | Signal peptidase I | 211 |
| O51389 | *BB*_0428 | unreviewed | Uncharacterized protein | 105 |
| Q44754 | *BB*_0266 | reviewed | Uncharacterized protein *BB*_0266 (ORF38) | 100 |
| O51266 | *BB*_0250 | reviewed | Inner membrane protein *BB*_0250 | 204 |
| O51234 | pstC *BB*_0216 | unreviewed | Phosphate ABC transporter, permease protein PstC | 302 |
| O51203 | *BB*_0185 | unreviewed | Glycoprotease family | 217 |
| O51178 | *BB*_0156 | unreviewed | Uncharacterized protein | 144 |
| O51140 | rpsR *BB*_0113 | reviewed | 30S ribosomal protein S18 | 96 |
| O51035 | *BB*_0001 | reviewed | Uncharacterized protein *BB*_0001 | 190 |
| O51706 | *BB*_0765 | unreviewed | Uncharacterized protein | 348 |
| O51663 | pgsA *BB*_0721 | unreviewed | CDP-diacylglycerol--glycerol-3-phosphate 3-phosphatidyltransferase | 209 |
| O51445 | rpsH *BB*_0492 | reviewed | 30S ribosomal protein S8 | 132 |
| O51368 | manA *BB*_0407 | unreviewed | Mannose-6-phosphate isomerase, class I | 372 |
| O51359 | *BB*_0398 | unreviewed | **Lipoprotein, putative** | 343 |
| O51318 | gatC *BB*_0343 | reviewed | Glutamyl-tRNA(Gln) amidotransferase subunit C | 91 |
| O51205 | *BB*_0187 | unreviewed | Uncharacterized protein | 86 |
| O51156 | *BB*_0130 | unreviewed | Uncharacterized protein | 255 |
| P0CL68 | dbpB *BB*_A25 | reviewed | **Decorin-binding protein B** | 187 |
| O51766 | *BB*_0826 | unreviewed | Uncharacterized protein | 171 |
| O51611 | *BB*_0667 | unreviewed | Uncharacterized protein | 183 |
| O51404 | *BB*_0448 | unreviewed | Phosphocarrier protein HPr | 91 |
| O51230 | *BB*_0212 | unreviewed | Borrelia ORF-A superfamily | 344 |
| O51736 | *BB*_0796 | unreviewed | Uncharacterized protein | 177 |

*Annotation table created by querying the UniProtKB database[75]. Status is defined by UniProtKB: “reviewed” indicates manually annotated records and “unreviewed” indicates automatically annotated records.

** **Bold** protein names indicate potential antigens.
