# Supplementary material for: Vectors as Epidemiological Sentinels: Patterns of Within-Tick Borrelia burgdorferi Diversity
Source: PLoS Pathog. 2016 Jul 14;12(7):e1005759. doi: 10.1371/journal.ppat.1005759 (PMC4944968; doi:10.1371/journal.ppat.1005759)
Supplement: S1 Text — (DOCX) [file ppat.1005759.s012.docx]

**S1 Text.** **Intrahost variant identification.**

*Sensitivity and specificity of intrahost variant identification.* A major challenge in minority variant analysis arises from the fact that minority variants often exist at low frequencies close to rate of frequencies introduced through Illumina sequencing and read mapping errors[20]. Though *Bb* chromosomes are highly syntenic, structural rearrangements (including attachment of plasmids) may introduce mapping errors[76,77]. Therefore, we used stringent filters for identifying minority variants and developed threshold for sequencing/mapping error described in the Methods. We evaluated the sensitivity and specificity of minority variant identification for both *in silico* single infections (Methods) as well as *in silico* mixed infections.

For *in silico* single infections, specificity (the probability that invariant sites are not identified as iSNVs is > 99.99%. (Sensitivity cannot be measured as there are 0 iSNVs in the simulated single infections) (Fig. S1).

To determine the sensitivity and specificity of our approach to detect within-host polymorphisms, we pooled reads simulated from two different *Bb* reference genomes (N40 and JD1) to generate *in silico* mixed infections. Reads were selected randomly from the two read-sets without replacement to generate mixed sequence data with the minority genome comprising 50, 40, 30, 20, 10, 5, 2.5, and 1 % of reads. Genome coverage (including reads from both samples) was held at 100X. Intrahost variants were identified as described in the Methods.

For each artificial mixture, the sensitivity (true positive rate) of intrahost variant calling is the proportion of positives (true SNP sites between the two mixed genomes) correctly identified by our minority variant caller (Fig. S2a). Specificity (true negative rate) is the proportion of negatives (conserved sites) correctly identified as such (Fig. S2b). True SNPs between the two genomes were identified with MUMMER[78]. For *in silico* mixed infections sequenced at 100X coverage, sensitivity (the probability that individual iSNVs are identified) is 57.37% for iSNVs present at minor allele frequencies (MAF) of 10%; sensitivity increases rapidly with MAF (Fig. S2). Specificity within mixed infections again is > 99.99% (Fig. S3).

*Comparing iSNV rates.* To compare intrahost variation between samples sequenced at different coverage levels, we calculated the rate of iSNVs for each site called at 40X coverage, as described in[26]. To determine the sequencing coverage (N) required to detect iSNVs, we modeled the probability of calling an iSNV (power) at a site with a given minor allele frequency (MAF), where the minor allele was present on at least 5 reads, with a negative binomial distribution. To call iSNVs with 90% power, N ≥ 130 for MAF of 5 %, N ≥ 62 for MAF of 10%. To call iSNVs with 60% power, N ≥ 80 for MAF of 5 %, N ≥ 39 for MAF of 10%. For Fig. 2B, we determine the iSNV rate including only sites called at 40X coverage (i.e. sites with sufficient coverage to call iSNVs at MAF of 10% with 60% power).
